# Thematic analysis of the medical records of patients evaluated for kidney transplant who did not receive a kidney

**DOI:** 10.1186/s12882-020-01951-1

**Published:** 2020-07-25

**Authors:** Catherine R. Butler, Janelle S. Taylor, Peter P. Reese, Ann M. O’Hare

**Affiliations:** 1 Division of Nephrology, Department of Medicine and the Kidney Research Institute, University of Washington, 1959 NE Pacific St, Campus Box 356521, Seattle, WA 98195 USA; 2 Department of Anthropology, University of Toronto, Toronto, Canada; 3 Renal-Electrolyte & Hypertension Division and Department of Biostatistics, Epidemiology, and Informatics, Perelman School of Medicine at the University of Pennsylvania, Philadelphia, PA USA; 4 Nephrology Section, Hospital and Specialty Medicine and Seattle-Denver Health Services Research and Development Center of Innovation, Veterans Affairs Puget Sound Health Care System, Seattle, Washington USA

**Keywords:** Kidney transplant evaluation, Person-centered medicine, Shared decision-making, End-stage kidney disease, Transplant, Qualitative analysis

## Abstract

**Background:**

A potential pitfall of policies intended to promote referral for kidney transplant is that greater numbers of patients may be evaluated for transplant without experiencing the intended benefit of receiving a kidney. Little is known about the potential implications of this experience for patients.

**Methods:**

We performed a thematic analysis of clinician documentation in the electronic medical records of all adults at a single medical center with advanced kidney disease who were referred to the local transplant coordinator for evaluation between 2008 and 2018 but did not receive a kidney.

**Results:**

148 of 209 patients referred to the local kidney transplant coordinator at our center (71%) had not received a kidney by the end of follow-up. Three dominant themes emerged from qualitative analysis of documentation in the medical records of these patients: 1) Forward momentum: patients found themselves engaged in an iterative process of testing and treatment that tended to move forward unless an absolute contraindication to transplant was identified or patients disengaged; 2) Potential for transplant shapes other medical decisions: engagement in the transplant evaluation process could impact many other aspects of patients’ care; and 3) Personal responsibility and psychological burden for patients and families: clinician documentation suggested that patients felt personally responsible for the course of their evaluation and that the process could take an emotional toll on them and their family members.

**Conclusions:**

Engagement in the kidney transplant evaluation process can be a significant undertaking for patients and families and may impact many other aspects of their care. Policies to promote referral for kidney transplant should be coupled with efforts to strengthen shared decision-making to ensure that the decision to undergo transplant evaluation is framed as an explicit choice with benefits, risks, and alternatives and patients have an opportunity to shape their involvement in this process.

## Background

Kidney transplant is the preferred treatment for many patients with advanced kidney disease [1, 2], but the limited availability of donor organs means that not all patients who might benefit can receive a kidney. In order to increase access to kidney transplant and promote more equitable organ allocation, a number of nationwide initiatives are currently underway to encourage transplant referrals [3]. The Centers for Medicare and Medicaid Services (CMS) requires that patients on dialysis be educated about transplant as a treatment option [4] and has incorporated dialysis facility transplant rates into some quality metrics [5–7]. Other national and regional initiatives focus on educating patients with advanced kidney disease about the option of kidney transplant and encouraging living kidney donation [8–11]. Increasing access to kidney transplant among patients with advanced kidney disease is also a central objective of the recent presidential Executive Order on Advancing American Kidney Health [12].

Given the relatively limited supply of donor kidneys, policies intended to promote more widespread and equitable access to kidney transplant may have the unintended effect of increasing the number of patients who undergo evaluation for kidney transplant but do not ultimately receive a kidney. The medical and psychosocial evaluation process required for kidney transplant can be extensive, and may entail multiple clinic visits with specialists, diagnostic tests (e.g., cancer screening, cardiac angiography), and behavior modification (e.g., stopping tobacco use, improving diet) [13]. In order to gain insight into potential unintended consequences of policies to increase transplant referral, we conducted a qualitative analysis of the electronic medical records of patients who were evaluated for kidney transplant but did not receive a kidney.

## Methods

### Study design and cohort selection

We conducted a thematic analysis of documentation in the electronic medical records of patients referred to the kidney transplant coordinator at the Veterans Affairs Puget Sound Health Care System (VAPSHCS) in Seattle, WA over a ten-year period who had not received a kidney by the end of follow-up. The transplant coordinator at our medical center receives referrals for veterans with advanced kidney disease from providers practicing both within and outside the Veterans Affairs Health Care System (VA). The coordinator’s role is to provide early education around transplant, organize and facilitate initial steps in the evaluation process, refer potentially eligible patients to a regional transplant center, and coordinate post-transplant care. The transplant program at the VA medical center in Portland, Oregon serves as the primary referral center for the VAPSHCS, but patients may also be referred to other VA and non-VA transplant centers.

**Fig. 1:**
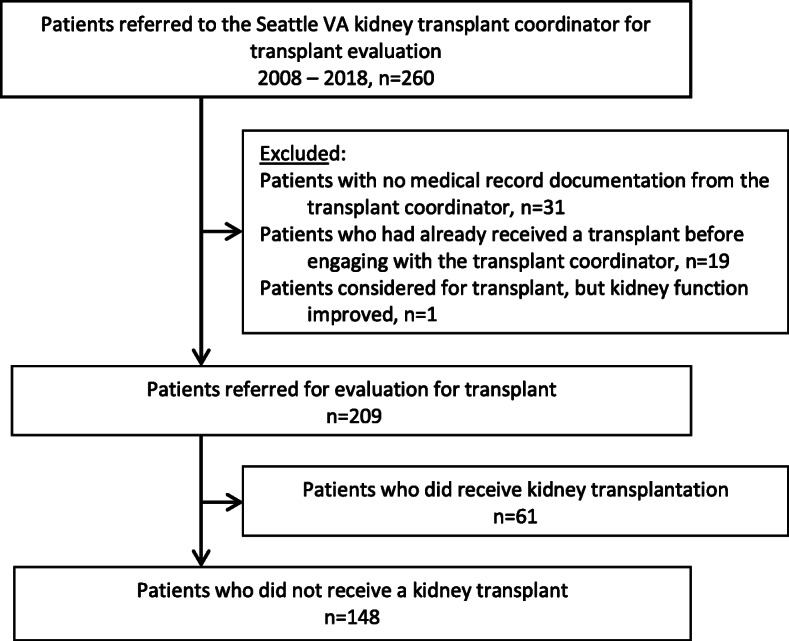
Flow-chart showing cohort derivation. Abbreviations: VA, Veterans Affairs Health Care System

Between January 1, 2008 and January 1, 2018, 260 patients were included in a comprehensive clinical registry of referrals to the local transplant coordinator (Fig. 1). We excluded patients who had already received a transplant when they first interacted with the transplant coordinator (*n* = 19), those whose kidney function subsequently improved (n = 1), and those who were referred to the transplant coordinator but did not follow up with her either in person or by phone (*n* = 31). Among the remaining 209 patients referred for transplant evaluation, we further excluded those who received a kidney transplant during follow-up based on information in the local registry and/or electronic medical record (*n* = 61). Because our goal was to gain a broad perspective on the entire transplant evaluation process across multiple care settings for those who did not receive a kidney, patients were included regardless of how far they had progressed in the evaluation process (e.g., initial discussion, referral to a transplant center, waitlisting).

The analytic cohort for this study consisted of the 148 patients (71% of all patients who were referred to the VAPSHCS transplant coordinator for transplant evaluation) who were evaluated for kidney transplant but had not received a kidney as of January 2018. The VAPSHCS Institutional Review Board approved this study and waived the requirement to obtain patients’ informed consent.

### Data collection

Each VA medical center maintains a comprehensive electronic medical record that includes all clinician notes for patients receiving care at that center. The electronic medical record system also includes a platform for remotely accessing patients’ records at other VA medical centers. One author (C.R.B. a senior nephrology fellow) reviewed the electronic medical records of all patients included in the registry to collect information on their age, sex, race, whether they were receiving dialysis at the time of first contact with the transplant coordinator, and whether they died during follow-up. Then, using a text search application embedded in the local electronic medical record to identify all mentions of the term “transplant” (which included all words with this root, e.g., transplants, transplanted, transplantation), she abstracted documentation pertaining to the transplant evaluation for each cohort member (both before and after referral to the transplant coordinator). She also manually reviewed all remote documentation for cohort members seen at regional VA transplant centers.

### Analyses

Using inductive thematic analysis, an approach to analyzing text that facilitates discovery of previously unidentified concepts related to a phenomenon of interest [14], two investigators experienced in qualitative methodology (C.R.B. and A.M.O., a nephrologist at the Seattle VA) independently reviewed all abstracted passages from the electronic medical records of a random selection of 50 cohort members who had not received a transplant. We coded for concepts relevant to the transplant evaluation until we reached saturation, or the point at which no new concepts were identified with additional coding [15, 16]. C.R.B. then reviewed and coded all abstracted passages from the remaining 98 patients who were referred for transplant evaluation but did not receive a kidney during follow-up. Together, these two investigators iteratively reviewed codes to identify emergent themes, returning as needed to the primary passages to resolve differences in interpretation and ensure that emergent themes were grounded in the data [14, 16]. The two other coauthors (J.S.T. a medical anthropologist and P.P.R. a transplant nephrologist) then reviewed emergent themes and exemplar quotations. All four authors together developed the final thematic schema. The local transplant coordinator was consulted for member checking of the final schema and to address any privacy concerns she might have related to inclusion of illustrative quotations from her own chart notes. We used SPSS, version 19 (IBM SPSS, Chicago, IL), for descriptive statistics and Atlas.ti version 8 (Scientific Software Development GmbH) to organize and store text and support initial coding and comparison across coders.

## Results

Overall, 148 patients (71%) who were referred to and interacted with the local transplant coordinator from 2008 to 2018 had not received a kidney by the end of follow up. Their mean age at the time of first interaction with the transplant coordinator was 61.2 years (standard deviation (SD) 7.9 years) (Table 1). All 148 patients were male, 18.9% were Black, 56.8% were White, and cche end of follow-up.

**Table 1:** Characteristics of patients who were evaluated for transplant, but did not receive a kidney, 2008–2018

	Patients (*n* = 148)
Age at initiation of transplant evaluation, y, (mean [SD])	61.2 (7.9)
Sex, (%)	
Male	148 (100.0)
Race, (%)	
Black	28 (18.9)
Asian	11 (7.4)
Native Hawaiian or Pacific Islander	9 (6.1)
Native Alaskan or American	2 (1.4)
White	84 (56.8)
Unknown or declined to report	14 (9.5)
Ethnicity, (%)	
Hispanic or Latino	5 (3.4)
Not Hispanic or Latino	131 (88.5)
Unknown or declined to report	12 (8.1)
ESKD at initiation of transplant workup, (%)	59 (39.9)
Year of initiation of transplant work up, (%)	
2008–2009	75 (50.7)
2010–2011	36 (24.3)
2012–2013	21 (14.2)
2014–2015	10 (6.8)
2016–2018	6 (4.1)
Died during the follow up period	122 (82.4)

*Abbreviations*: *SD* standard deviation, *ESKD* end-stage kidney disease

### Qualitative analysis

The following three overlapping and interrelated themes emerged through inductive thematic analysis of clinician documentation in patients’ electronic medical records: 1) Forward momentum; 2) Potential for transplant shapes other medical decisions; and 3) Personal responsibility and psychological burden for patients and families.

#### Theme 1. Forward momentum

Patients who were referred to the transplant coordinator found themselves engaged in an ongoing and iterative process of transplant evaluation that tended to move forward until an absolute contraindication was identified or the patient disengaged (Table 2).

**Table 2:** Forward momentum (Theme 1)

Subtheme	Illustrative Quotations (*source*)
The evaluation process proceeds reflexively	Debilitated man who is blind, deaf, and requires a wheel chair … His creatinine today is 4.0, so I think he should be referred for fistula evaluation … We also discussed the possibility of transplant, which seems unlikely, but I will mention him to the transplant team. (*nephrologist)* [The patient] was declined as a kidney transplant candidate … However, they also said that “If he is able to resolve his peripheral vascular disease issues you could re refer him to [the transplant center] again at a later date.” (*nephrologist*) Kidney transplant was “DECLINED” because the team felt patient was high risk candidate [due to] co-morbid conditions … If Medicare supplement insurance is available we could refer this patient on to [a second transplant center]. (*transplant coordinator*) [The patient] is undecided about transplant saying he needs to think about it. Worried that he would be “taking a kidney away” from a younger person … I currently see no contraindication to transplant for this Veteran. (*transplant coordinator*) I have left a number of phone messages for [the patient] regarding completion of his pre-transplant evaluation. To date he has not returned any of my calls. (*transplant coordinator*)
A step-wise and piecemeal approach to testing and treatment	I spent 40 min talking with [the patient] about his declining health (… poor functional capacity) in the context of his candidacy for renal transplant. Nonetheless, [the patient] is determined to move forward with cardiac catheterization as recommended by cardiologist. (*transplant coordinator*) Our plan of attack will be to start the evaluation with the issues most likely to represent a barrier to transplant. (*transplant coordinator*) Cigarette smoking is an absolute barrier to transplant. You will need to be smoke free for at least a few months before we could consider starting a pre-transplant evaluation. (*transplant coordinator*) There is scintigraphic evidence of a small area of mild myocardial ischemia … a consult has been placed to cardiology. (*nuclear medicine physician*) … [Seen cardiology and now] post stent … and request that patient be further evaluated via myoperfusion study. Additionally a left “cervical” bruit was noted and thus a carotid duplex was requested. (*transplant coordinator*) He understands it takes time/is a slow process, but he said “tests keep being forgotten, and when I’m just about ready to get on the list, they remember they forgot another test.” (*social worker*)
Uncertainty about what to expect from the evaluation process	Patient states that he may have been placed on the renal transplant list, but unable to clarify status at this time … States that he has an appointment coming up next week. I also asked the patient to clarify his renal transplant status at this time. I will have him come back to the clinic in four weeks with this updated information and make plans for possible knee replacement. (*orthopedic surgeon*) He wanted to know when he’ll be having surgery (kidney transplant). I reminded [the patient] that his referral was deferred by [the transplant center] and that he must FIRST be seen at his transplant center and accepted as a patient before he will be listed for [deceased donor] renal transplant. (*transplant coordinator*) The patient also continues metoprolol 25 mg twice a day and atorvastatin 20 mg a day for hypercholesterolemia. He also asked me how this would affect his ability to get back on the renal transplant list. I told him I really did not know and he should address this with his nephrologist. (*cardiologist*) Gentleman with chronic hepatitis C … liver biopsy would be indicated to sort this out as it might change plans in terms of renal transplant. The couple wanted to know more about this and I asked them to talk to their Nephrologist or [transplant coordinator] about in the event that we find cirrhosis would that disqualify him for a renal transplant, as I was not clear on the answer. (*hepatologist*)

Non-standard medical abbreviations have been expanded and typographical errors corrected to improve clarity and readability

##### The evaluation process proceeds reflexively.

Once initiated, the evaluation process tended to move forward unless there was a clear absolute medical, psychosocial, or behavioral contraindication to kidney transplant. Even when there was a recognized contraindication, clinicians might mention the possibility of resuming the evaluation process at a future time if the relevant barrier(s) could be addressed. Sometimes, patients were referred to a second transplant center after being declined by the primary center.

Patients’ ambivalence about proceeding with the transplant evaluation seemed less likely to halt the evaluation process than the presence of an absolute medical contraindication. It was rare for patients to make an overt decision to stop the evaluation in the absence of a clear contraindication. More commonly, we saw what appeared to be a passive process of disengagement whereby patients simply did not follow through with recommended clinic visits, testing, or treatments without any chart documentation to suggest that there had been an explicit decision not to pursue transplant.

##### A stepwise and piecemeal approach to testing and treatment

The transplant evaluation tended to be organized by organ system with a focus on identifying markers of disease severity that would constitute an absolute contraindication to kidney transplant. Components of the evaluation process were typically conducted in sequence, rather than in parallel, so that “major” contraindications would be identified early on. This stepwise approach to evaluation could be a source of frustration for some patients who, after successfully surmounting one barrier, might be surprised to learn that more testing lay ahead. Abnormal test results could also trigger a cascade of follow-up testing. While poor overall health, frailty, and functional limitations might be documented as concerning, these more global measures were rarely seen as absolute contraindications to transplant.

##### Uncertainty about what to expect from the evaluation process

Documentation in patients’ electronic medical records suggested that both they and their local providers were often uncertain about what the evaluation process might entail and about the requirements for transplant. Providers who were more peripherally involved in the evaluation process might rely on patients for information about the status of their candidacy. Subspecialists responsible for discrete components of the evaluation tended to assume a more technical role, deferring interpretation of test results to the transplant coordinator, nephrologist, or transplant team.

#### Theme 2. Potential for transplant shapes other medical decisions

The transplant evaluation unfolded in the broader context of patients’ other health conditions and evolving course of illness and could impact many other aspects of their care (Table 3).

**Table 3:** Potential for transplant shapes other medical decisions (Theme 2)

Subtheme	Illustrative Quotations (*source*)
Exposing and treating subclinical conditions	Patient has no symptoms referable to angina and has a good functional capacity … does not have a good clinical indication for PCI. However, if his transplant work-up deems it absolutely necessary, then PCI could be considered. (*cardiologist*) Recommend extraction of [tooth] #1 due to gross decay. There is a low risk of tooth becoming abscessed due to level of decay... Patient did not want extraction at this time. Patient advised dental clearance [for transplant] will not be given until tooth is removed. (*dentist*) As part of the [transplant] work up he was noted to have new elevated left hemidiaphragm for which a CT scan of the chest was performed. He was noted to have dilated pancreatic duct with multiple pancreatic calcifications and was sent here for further work up. (*gastroenterologist*) … He underwent endoscopic ultrasound and endoscopic retrograde ERCP to further evaluate for evidence of cancer. Approximately 1 h after the procedure, he started to report right upper-quadrant pain...consistent with post-ERCP pancreatitis. (*hospitalization discharge summary)*
Decisions about dialysis and transplant are interdependent	His goal to “avoid” dialysis may become his stimulus to learn more [about transplant]. (*psychiatrist*) Patient adamant that he does not want dialysis, discussed that given his rate of decline in GFR he may need renal replacement therapy soon, hopefully as a bridge to transplant. (*nephrologist*) [The patient’s wife] tells me that [the patient] is still working as well as going for HD 3 times per week. The family is feeling overwhelmed “we’re doing the best we can”. (*transplant coordinator, calling to inquire about the reason for delayed transplant clinic visits*) Request that he have cardiac catheterization prior to [transplant center] approval/denial for transplant. [The patient] understands that this procedure may negatively impact his kidneys and force him to begin dialysis. (*transplant coordinator*)
Transplant evaluation shapes other aspects of care	Recommend repeating vaccination series. Patient skeptical of this as he doesn’t want it to affect his upcoming transplant. (*gastroenterologist*) [The patient’s wife] reports that he had significant hesitation to seek treatment for his depression because he believed that he would be removed from the kidney transplant list if they found out he was being treated. (*psychiatrist*) The Veteran … has a long history of left knee osteoarthritis, which is debilitating to him (*transplant center note*) … It doesn’t seem that it would be best for him to have a knee replacement now when he is high priority for a kidney and this may disrupt his place on the waiting list. (*orthopedic surgeon*) Lymphadenopathy was incidental finding on non-contrast MRI completed [years ago] … In light of this patient’s interest and desire for kidney transplant this issue must be fully explored and malignancy ruled out … He told me “I don’t want to do any more tests’ … he understands the possible consequences (progression of a yet to be diagnosed disease/cancer), “I don’t want to know if there’s something wrong.” (*transplant coordinator*) The transplant team told him that he would not be a candidate for transplant because he was using a wheelchair for his mobility … he said that he was determined to walk so he could be considered for transplant. (*psychiatrist*) [Primary nephrologist] felt that by being motivated by potential transplant he may be more compliant...He needs to show compliance with weight modifications and blood pressure. Will make sure he is controlled before placement on list. (*transplant center note*)

Non-standard medical abbreviations have been expanded and typographical errors corrected to improve clarity and readability

*Abbreviations*: *PCI* percutaneous coronary intervention; *CT* computed tomography scan, *ERCP* cholangiopancreatography, *GFR* glomerular filtration rate, *HD* hemodialysis, *MRI* magnetic resonance imaging

##### Exposing and treating subclinical conditions

A variety of asymptomatic conditions might be identified during the transplant evaluation process (e.g., subclinical coronary artery disease, hepatic fibrosis, mild cognitive impairment). Detection of these abnormalities could prompt further testing and interventions not ordinarily indicated as part of routine care.

##### Decisions about transplant and dialysis are interdependent

Kidney transplant was sometimes framed as a way of avoiding dialysis or dialysis might be characterized as a bridge to transplant. Cardiac catheterization—which was a key component of the transplant evaluation for many patients—often raised concerns about precipitating the need for dialysis. For those on dialysis, the demands of their treatment schedule could make it difficult to participate in the transplant evaluation.

##### Transplant evaluation shapes other aspects of care

Patients and clinicians sometimes avoided treatment for symptomatic conditions (e.g., mood disorders, osteoarthritis) due to concerns about the potential implications this might have for transplant candidacy (e.g., by exposing psychological vulnerabilities or precipitating clinical deterioration). In other cases, tests and procedures required for the transplant evaluation might carry potential for harm or conflict with patients’ other goals and preferences. It was common to see medical treatments, diagnostic tests, and recommended behavioral modifications—many with potential health benefits in their own right—viewed by patients and providers through the narrow lens of how these might shape transplant candidacy.

#### Theme 3. Personal responsibility and psychological burden for patients and families

Electronic medical record documentation suggested that patients could feel personally responsible for the course of the transplant evaluation, and that the process could take an emotional toll on them and their families (Table 4).

**Table 4:** Personal responsibility and psychological burden for patients and families (Theme 3)

Subtheme	Illustrative Quotations (*source*)
Responsibility for becoming a “good candidate”	Patient states that he wants to “prove to everyone” that he can do what is necessary to be a good peritoneal dialysis candidate as well as a transplant candidate. (*nutritionist*) Must encourage self-determination and responsibility for performing the suggested dental work to avoid infection before can be activated on transplant list. (*dialysis unit nurse*) Admitted that … he had indicated he had stopped smoking (which he had not) [the patient] appeared truly sorry and upset. (*dialysis unit nurse*) Being a loner is not a good style for transplant, so the veteran will benefit from learning to reach out & be more inclusive during the phases of transplant *(psychiatrist*) The [transplant center] team wants him to be less dependent on his mother and asked that he go to vocational rehab to learn job skills … I encouraged [the patient] to consider volunteering as a start. (*transplant coordinator*) Discussed patient’s current lack of compliance with meds, blood sugar readings, etc. Discussed how this continued non-compliance does not make him a good candidate for transplant, because the regimen he needs to maintain post-transplant is much more demanding. (*social worker*) Patient has NOT BEEN taking all medications as ordered. Reminded patient of importance of taking medications and that compliance with therapies will be noted by the transplant workup staff and those who will evaluate his ability to work with team for transplant. Patient indicates that he really wants to make this work and promises to make a more concentrated effort. (*dialysis unit nurse*) His coping skills, level of family support, and compliance will be tested once he starts dialysis, and we will be able to better assess these concerns at that time. (*nephrologist*)
Anxiety and psychological distress	Transplant workup was begun … however he and his family have decided that “it’s just too much...”, too many appointments, too much “back and forth”. (*transplant coordinator*) Patient and [his wife] were not getting along today. This is the first time this social worker has ever witnessed this ...it is obvious that the pressure and stress of this has affected both patient and [his wife]. (*social worker, transplant evaluation note*) It was clear that from a psychological perspective a backup person for [peri-transplant] caregiver was very important for this veteran & his spouse. His spouse was feeling overwhelmed and had panic attacks. (*psychiatrist*) Does seem somewhat anxious about all the appointments he has for his transplant work-up … becomes very anxious if there are changes or deviations in the process. (*social worker*) [The patient] has a long history of major depression with multiple episodes in the past several years...sudden “crash” 2 weeks prior when learning that his brother would not be able to donate kidney. (*psychiatrist*) Veteran primarily expressed feelings of anger; he sees this decision [rejection from the transplant center] as arbitrary made by “some bureaucrats”. (*psychiatrist*) He had a history of three arrests, three incarcerations...history of probation & history of parole … [patient] said that he was saddened by his mis-steps & behaviors which results in the arrest/incarceration … He said that he was very sorry for behaving so bad & he said that talking about it was embarrassing for him. (*psychiatrist, transplant evaluation note*)

Non-standard medical abbreviations have been expanded and typographical errors corrected to improve clarity and readability

##### Responsibility for becoming a “good candidate”

Chart documentation suggested that both patients and clinicians believed that personal motivation, willing engagement in the evaluation process, and adherence to treatment recommendations were essential if patients were to receive a kidney. Clinicians’ notes captured patients’ expressions of guilt and remorse about health factors and behaviors that might present barriers to transplant.

Documentation in patients’ medical records suggested that clinicians expected patients to assume primary responsibility for managing their medical conditions and addressing behavioral barriers to transplant candidacy. How patients conducted themselves during the transplant evaluation—and especially how they coped with dialysis treatment—tended to be viewed as a marker for how they would fare after transplant. Clinicians sometimes recommended changes to patients’ personal and social lives in order to strengthen their candidacy for transplant. Family members could also find themselves tasked with new responsibilities such as organizing a complex schedule of appointments and transportation.

##### Anxiety and psychological distress

The transplant evaluation process could have a deleterious psychological and emotional impact on patients and family members. The large number of required appointments and tests, scheduling, and transportation arrangements could be overwhelming. The transplant psychosocial evaluation could also be perceived as intrusive and/or embarrassing for some patients. Abrupt changes in expectations about candidacy, setbacks related to newly diagnosed health conditions, and news that transplant candidacy had been declined were documented as sources of disappointment, distress, and anxiety for patients and families. Clinicians’ notes also conveyed patients’ uncertainty about what to expect from the transplant evaluation process and poor understanding of selection criteria and rationale for selection decisions.

## Discussion

Most patients evaluated for kidney transplant at our center from 2008 to 2018 did not receive a kidney. Our analyses suggest that engagement in the transplant evaluation process could take a substantial physical and emotional toll on these patients and their family members and impact many other aspects of their care. These findings suggest that policies to promote referral for transplant should be accompanied by efforts to strengthen shared decision-making to ensure that referral is framed as an explicit treatment choice with benefits, risks, and alternatives.

Despite the substantial tradeoffs involved, the transplant evaluation process tended to move forward in a somewhat reflexive fashion with few opportunities for patients to modulate their involvement short of simply not following through with clinicians’ recommendations. Testing undertaken as part of the evaluation could lead to a cascade of additional tests and treatments that would not ordinarily have been recommended as part of routine care, while an incremental and piecemeal approach to the evaluation process could also make it difficult for patients to anticipate what might lie ahead [17, 18]. This kind of forward clinical momentum—which has been described in a number of other clinical contexts—can limit opportunities for shared decision-making [19–22]. Not only can the promise of longevity and better quality of life through kidney transplant be difficult to resist [23–25] but the pursuit of transplant can become an all-consuming goal for many patients, with far-reaching impacts on other aspects of their care [26].

While it is appropriate that the transplant evaluation and selection process are shaped by societal considerations beyond the needs of individual patients, local clinicians and transplant center teams still have an obligation to support the values, goals, and preferences of patients and families to the greatest extent possible. Although many patients were highly motivated to receive a kidney, the evaluation process itself could be burdensome and emotionally taxing and could have unintended effects on many other aspects of care. Not only did patients experience disappointment at not receiving a kidney, but they might also feel morally responsible for the course of the evaluation [24, 27]. In light of these tradeoffs, our findings suggest that evaluation for transplant should be framed not simply as a preliminary step toward receiving a kidney, but as an explicit treatment choice in its own right [17, 28–30]. This may be especially important for older adults and/or those with multiple comorbidities, many of whom are both more susceptible than their younger counterparts to the unintended harms of the evaluation process and less likely to ultimately benefit by receiving a kidney [18, 31].

Our findings resonate with prior work describing how poor communication [32] and lack of clarity about what to expect from the transplant evaluation process among patients and their local clinicians [33–35] can serve as barriers to shared decision-making. Most prior work in this area has focused on later steps in the transplant process such as wait-listing, transplant surgery, and life after transplant [36–40]. Our findings add to this literature by suggesting that stronger efforts are needed to support shared decision-making early in the transplant evaluation process, including at the time of referral. This might include more explicit discussions about what the evaluation and selection process typically involves, consideration of patients’ hopes, expectations, and big-picture health priorities, and engagement of family members in the decision-making process [41, 42]. More work to elicit the perspectives of patients and families at different stages in the transplant evaluation process could also inform efforts to support a more person- and family-centered approach to referral decisions.

The availability of a comprehensive registry of patients referred for transplant evaluation at our center and the integrated nature of our medical system offered a rare window on the formative steps in the evaluation process and episodes of care occurring across a range of clinical settings that can otherwise be difficult to capture. This approach can be helpful in understanding complex care processes and treatment decisions that are well documented in the medical record. Nonetheless, our results should be interpreted with the following limitations in mind. First, the care processes described in this single-center study may not be directly relevant to patients cared for at other centers within or outside the VA. Second, our results may not describe care processes for groups of patients who are poorly represented in our study cohort (e.g., women and young adults). Because our goal was to identify themes relevant to patients who were evaluated for kidney transplant and did not receive a kidney and most (82.4%) of those included in the study died during follow-up, our findings are not intended to be generalizable to patients who complete the evaluation process and go on to receive a kidney. Third, because our goal was to identify themes relevant to the entire evaluation process, we did not distinguish between patients based on whether they were referred to a regional transplant center or added to the deceased donor waitlist. Finally, our analysis was based on clinician documentation in patients’ electronic medical records, and thus cannot fully describe the experiences and perspectives of patients, families, or clinicians.

## Conclusion

The great majority of patients evaluated for transplant at our center from 2008 to 2018 did not receive a kidney. The themes that emerged from qualitative analysis of clinician documentation in the electronic medical records of these patients suggest that engagement in the transplant evaluation process can be a major undertaking for patients and families, can impact many other aspects of patients’ care, and offers few opportunities for patients to actively shape the process. These findings suggest that policies intended to increase referral for kidney transplant should be coupled with efforts to strengthen shared decision-making to ensure that this is framed as an explicit choice with potential benefits, risks, and alternatives.

## Data Availability

The data that support the findings of this study were obtained from patients' electronic medical records and are not publicly accessible.

## Abbreviations

CMS: Centers for Medicare and Medicaid Services
ESKD: End-stage kidney disease
UNOS: United Network for Organ Sharing
VAPSHCS: Veterans Affairs Puget Sound Health Care System
VA: Veterans Affairs Health Care System
SD: Standard deviation
